# Rasch fit statistics and sample size considerations for polytomous data

**DOI:** 10.1186/1471-2288-8-33

**Published:** 2008-05-29

**Authors:** Adam B Smith, Robert Rush, Lesley J Fallowfield, Galina Velikova, Michael Sharpe

**Affiliations:** 1 Cancer Research UK – Clinical Centre, St. James's University Hospital, Leeds, UK; 2 Centre for Health & Social Care, University of Leeds, Leeds, UK; 3 Centre for Integrated Health Research, Queen Margaret University, Edinburgh, UK; 4 Psychosocial Oncology Group – Cancer Research UK, University of Sussex, UK; 5 School of Molecular & Clinical Medicine, University of Edinburgh, Edinburgh, UK

## Abstract

**Background:**

Previous research on educational data has demonstrated that Rasch fit statistics (mean squares and t-statistics) are highly susceptible to sample size variation for dichotomously scored rating data, although little is known about this relationship for polytomous data. These statistics help inform researchers about how well items fit to a unidimensional latent trait, and are an important adjunct to modern psychometrics. Given the increasing use of Rasch models in health research the purpose of this study was therefore to explore the relationship between fit statistics and sample size for polytomous data.

**Methods:**

Data were collated from a heterogeneous sample of cancer patients (n = 4072) who had completed both the Patient Health Questionnaire – 9 and the Hospital Anxiety and Depression Scale. Ten samples were drawn with replacement for each of eight sample sizes (n = 25 to n = 3200). The Rating and Partial Credit Models were applied and the mean square and t-fit statistics (infit/outfit) derived for each model.

**Results:**

The results demonstrated that t-statistics were highly sensitive to sample size, whereas mean square statistics remained relatively stable for polytomous data.

**Conclusion:**

It was concluded that mean square statistics were relatively independent of sample size for polytomous data and that misfit to the model could be identified using published recommended ranges.

## Background

Although Rasch Models [1] were originally designed and used for educational assessment in recent years they have increasingly been used in health research. This renewed interest in these models has largely been encouraged by a number of potential advantages of Rasch models over traditional psychometric methods, including the ability to decrease the number of items in questionnaires to reduce patient burden whilst retaining the psychometric properties of the instrument, and the pooling of data drawn from different samples allowing more accurate parameter estimation. Recent studies in health have explored the use of Rasch models in instrument development [2-4], modification of existing questionnaires [5-8], as well as in instrument and cross-linguistic comparison [9,10].

Rasch Models are a family of measurement models [11] which can be used to describe latent traits where items from questionnaires and person scores are located along the same scale of the latent trait. Item location ("difficulties") and person measures ("abilities") are estimated separately to produce estimates for each parameter which are sample and item independent respectively [12]. Rasch Models specify a number of criteria, which if fulfilled result in interval scales where adjacent scores along the scale are equally spaced, a feature which is particularly important for interpreting clinically meaningful differences [13]. Firstly, the data should describe a unidimensional construct, that is, a single latent trait should explain the variance in the data. The existence of dimensionality can be assessed using principal components analyses of the residuals [14]. Secondly, item invariance stipulates that item (or person) parameters should be independent of the sample (or items) used. This item invariance criterion can be evaluated using differential item functioning to determine whether item bias is present. The final criterion, which will form the focus of this paper, is item fit, in other words whether individual items in a scale fit the Rasch model.

There has been and there continues to be a considerable debate around the issue of which is the most appropriate fit statistic to use, what range of fit statistics to be employed when evaluating fit, and how fit statistics should be interpreted [15,16].

The use of chi-square statistics or infit and outfit mean squares to assess item fit to the model (described in more detail below) has been advocated. The mean squares can be converted through a cube-root transformation (Wilson-Hilferty) to (infit/outfit) t-statistics.

The mean square fit statistics are perhaps the most commonly used fit statistics in health research. A series of ranges has been suggested [17] to be employed when evaluating item fit depending on the type of test, however the majority of studies employ a range of 0.7 to 1.3. Despite the popularity of this approach some concerns have been voiced about the use of a single, universal range to evaluate fit and the lack of adjustment of the range to sample size. For instance, Smith et al. [16] using simulated datasets on dichotomous data have determined that Type I error rates (defined here as the probability of falsely rejecting an item as not fitting the Rasch model) were significantly less than α = 0.05 for both infit and outfit mean squares using a range of critical values (0.7, 0.8, 0.9 – 1.1, 1.2, 1.3). Furthermore, Type I error rates decreased for the outfit mean square as sample size was increased. In contrast, the Type I error rates for the t-statistics, although not equal to 5% demonstrated fewer discrepancies.

More recently, studies [18] have demonstrated using data collected from a large sample of examinees' results that t-statistics may potentially identify more items that do not fit the model than both the infit and outfit mean square fit statistics. For instance, the number of misfitting items identified by the t-statistic was four times greater than those identified by the mean square fit statistic (23 and 5, respectively).

In addition to research on the dichotomous model, recent work on the polytomous (Rating Scale) model with simulated data has suggested that the variability of mean squares is dependent on sample size and furthermore that the standard deviations for the t-statistics are generally smaller than their expected value (unity) [19]. These authors propose adjusting the critical range employed for both types of fit statistic depending on sample size.

Finally, Smith & Suh [18] have concluded that using mean square statistics may lead researchers to missing significant numbers of misfitting items, which may have an important impact on the development of unidimensional instruments, and that there is, furthermore, a need to understand Type I error rates associated with critical values for fit statistics. On the basis of this Smith and colleagues [16,18] have suggested that the t-statistic rather than the weighted and unweighted mean squares should be used to identify misfit, given that this statistic appears to be less sensitive to changes in sample size or alternatively to adjust mean square fit statistics using a correction based on the square root of the sample size [16].

However, despite this assertion there are a number of other methodological studies [15,20] which have shown that the t-statistic is highly sample dependent.

The evaluation and identification of item misfit is critical to the development of unidimensional instruments, and reliable fit statistics play an important part in this. There is uncertainty in the literature to assist health researchers in determining the most appropriate fit statistic to select for developing or modifying questionnaires. Previous research on simulated datasets has focused on the relationship between sample size and fit statistics at the level of groups of items. However, for test users the emphasis is more on which fit statistics are able to identify misfit consistently for individual items. Identification and removal of misfitting items will not only reduce patient burden, but may also improve person measure assessment [5].

Therefore the aim of this study was to investigate the impact of sample size on four commonly used fit statistics, i.e. infit/outfit mean square and their t-statistics for two polytomous Rasch models using data collected from a cancer patient sample.

The study attempted to determine: 1). whether fit statistics (and therefore Type I error rates, i.e. the probability of falsely rejecting an item which does fit the Rasch model) vary with sample size and 2). whether there were any differences in this variation between the different types of fit statistic.

## Methods

### Participants

Patient data were pooled from a number of studies carried out by Cancer Research – UK Psychological Medicine Group, Western General Hospital, Edinburgh (Scotland) over the past decade. The data have been collated from patients who completed a touchscreen version of both the HADS and the PHQ-9 in outpatient oncology clinics.

A total of 4072 patients completed the HADS (2781 females and 1291 males), and 3556 patients completed the PHQ-9 (2268 females and 1288 males). The average age of the sample was 60 years. Further clinical and demographic details are available from the published studies [21].

The studies from which these data have been drawn have all received ethical approval from the local research ethics committee.

### Instruments

#### The Hospital Anxiety & Depression Scale (HADS)

The Hospital Anxiety and Depression Scale (HADS) [22] was originally developed for screening for psychological distress in the general medical population. The scale consists of 7 items forming a Depression subscale (HADS-D), and 7 items forming an Anxiety subscale (HADS-A). Patients are asked to rate how they have felt in the past week on a 4-point scale (scored 0–3). It has been claimed that scores on the two subscales may also be summed to provide a total score (HADS-T), measuring psychological distress [23]. Previous research in a large heterogeneous cancer population [6] has shown potential misfit on three of the instruments' items: Anxiety 6 ("I get a sort of frightened feeling") and Depression 5 and 7 ("I have lost interest in my appearance" and "I can enjoy a good book, radio or TV programme" respectively). This misfit was present both in the full, 14-item version of the HADS as well as for the individual subscales. Although a principal components analysis of the residuals did not reveal the presence of any additional factors, given the misfitting items the analysis presented here will focus on the two subscales, HADS-Anxiety (A) and HADS-Depression (D).

#### Patient Health Questionnaire (PHQ-9)

The Patient Health Questionnaire – 9 (PHQ-9) [24] is a nine-item self-administered questionnaire which may be used for detecting and assessing the severity of depression. The instrument is based on the Diagnostic and Statistical Manual of Mental Disorders (DSM-IV) [25] criteria for diagnosing depression, and is scored on a 4-point scale ("not at all" to "nearly every day"). Patients are asked to rate any problems experienced over the last two weeks.

### Rasch Models

Both the Rating Scale and Partial Credit models are members of the family of Rasch Models [1]. The Rating Scale Model [26] is commonly employed to analyse Likert-type data [see Additional file 1]. As with all Rasch Models, the Rating Scale describes a probabilistic relationship between item difficulty (D) and person ability (B). In addition to this, thresholds are derived for each adjacent response category in a scale. In general, for *k *response categories, there are *k*-1 thresholds. Each threshold has its own estimate of difficulty (F_k_). The Rating Scale Model [see Additional file 1] describes the probability, P_ni _of a person with ability B_n_, choosing a given category with a threshold F_k _and item difficulty D_i_. A single set of thresholds is estimated for all items in a scale. The Partial Credit Model [27] can be seen as a modification of the Rating Scale Model where the threshold estimates are not constrained, that is, threshold estimates are free to vary between each item within a scale. Therefore for *N *items there will be *N*(*k *– 1) threshold estimates for the Partial Credit Model.

### Rasch Fit Statistics

Rasch fit statistics describe the fit of the items to the model. The mean square fit statistics have a chi-square distribution and an expected value of 1, where fit statistics greater than 1 can be interpreted as demonstrating more variation between the model and the observed scores, e.g. a fit statistic of 1.25 for an item would indicate 25% more variation (or "noise") than predicted by the Rasch model [11], in other words there is an underfit with the model. Conversely, an item with a fit statistic of 0.70 would indicate 30% less variation (or "overlap") than predicted or the items overfit the model. Items demonstrating more variation than predicted by the model can be considered as not conforming to the unidimensionality requirement of the Rasch model.

There are two commonly used mean square fit statistics, namely the infit mean square (also referred to as the weighted mean square) and outfit (or unweighted) mean square. Both mean squares are derived from the squared standardised residuals for each item/person interaction [see Additional file 1]. The outfit mean square is the average of the standardised residual variance across items and persons and is unweighted, meaning that the estimate produced is relatively more affected by unexpected responses distant to item or person measures. For the infit mean square the residuals are weighted by their individual variance (W_ni_) [see Additional file 1] to minimise the impact of unexpected responses far from the measure. The infit mean square is relatively more affected by unexpected responses closer to item and person measures [11].

The infit and outfit mean squares can be converted to an approximately normalised t-statistic using the Wilson-Hilferty transformation [see Additional file 1]. These infit/outfit t-statistics have an expected value of 0 and a standard deviation of 1. These statistics are evaluated against ± 2, where values greater than +2 are interpreted as demonstrating more variation than predicted.

### Method

The relationship between sample size and fit statistics was explored using the two Rasch models, that is, the Rating Scale Model [26], and Partial Credit Model [27]. The analysis was performed using *Winsteps *version 3.64 [14]. Eight sample sizes were used for each questionnaire: 25, 50, 100, 200, 400, 800, 1600, and 3200. Ten samples were drawn with replacement for each sample size for each item for the two instruments. Therefore, for the HADS there were 1120 data points (14 items × 8 sample sizes × 10 samples), and 720 data points for the PHQ-9 (9 items × 8 samples sizes × 10 samples). Ten samples were collated for each sample size (25 to 3600) for each questionnaire to produce an average for each of the four fit statistics (infit/outfit mean squares (MNSQ) and t-statistic (ZSTD in *Winsteps*)) for each item. This process was completed for both Rasch models.

## Results

### 1. Fit Statistics – Type I error rate

Tables 1, 2, 3, 4 show the fit statistics for each item averaged across sample size and provide an indication of the Type I error rates. For both the HADS subscales and PHQ-9 a Type I error rate of 5% would translate into approximately 1 misfitting item identified by chance alone.

**Table 1:** Fit statistics for the HADS subscale items (collapsed across sample size) for the Rating Scale Model

		**Infit MNSQ**	**SE**	**Infit t**	**SE**	**Outfit MNSQ**	**SE**	**Outfit t**	**SE**
	**ANX1**	0.92	0.04	-1.12	0.26	0.93	0.04	-1.07	0.26
	**ANX2**	1.02	0.02	0.11	0.17	0.98	0.02	-0.40	0.18
	**ANX3**	0.95	0.04	-1.06	0.25	0.95	0.04	-1.11	0.27
	**ANX4**	0.90	0.03	-1.38	0.28	0.97	0.04	-0.13	0.28
	**ANX5**	0.87	0.03	-1.71	0.23	0.88	0.04	-1.37	0.22
	**ANX6**	**1.46**	0.03	**5.92**	0.20	**1.46**	0.03	**5.97**	0.2
	**ANX7**	0.80	0.03	***-3.49***	0.33	0.76	0.03	***-3.71***	0.28
									
**Underfit**	**>1.30**	1.00	**>2**	1.00	**>1.30**	1.00	**>2**	1.00	
**Overfit**	**<0.70**	0	**<-2**	1.00	**<0.70**	0	**<-2**	1.00	

		**Infit MNSQ**	**SE**	**Infit t**	**SE**	**Outfit MNSQ**	**SE**	**Outfit t**	**SE**

	**DEP1**	1.02	0.04	-0.18	0.25	0.91	0.03	-1.56	0.21
	**DEP2**	1.09	0.06	1.35	0.31	0.97	0.07	0.01	0.25
	**DEP3**	0.86	0.06	-1.82	0.33	0.81	0.05	-1.72	0.27
	**DEP4**	1.03	0.03	0.51	0.22	1.10	0.04	1.65	0.32
	**DEP5**	**1.34**	0.07	**3.48**	0.29	1.18	0.09	1.17	0.22
	**DEP6**	0.77	0.03	***-3.06***	0.23	0.68	0.03	***-3.83***	0.19
	**DEP7**	**1.37**	0.07	**3.78**	0.29	**1.33**	0.07	**2.24**	0.18
									
**Underfit**	**>1.30**	2.00	**>2**	2.00	**>1.30**	1.00	**>2**	1.00	
**Overfit**	**<0.70**	0	**<-2**	1.00	**<0.70**	1.00	**<-2**	1.00	

**Table 2:** Fit statistics for the HADS subscale items (collapsed across sample size) for the Partial Credit Model

		**Infit MNSQ**	**SE**	**Infit t**	**SE**	**Outfit MNSQ**	**SE**	**Outfit t**	**SE**
	**ANX1**	0.97	0.04	-0.17	0.25	0.98	0.04	-0.06	0.27
	**ANX2**	0.88	0.03	-1.87	0.23	0.87	0.03	-1.78	0.22
	**ANX3**	0.86	0.03	***-2.41***	0.26	0.90	0.04	-1.89	0.28
	**ANX4**	1.05	0.03	0.95	0.23	1.06	0.04	1.12	0.24
	**ANX5**	0.92	0.03	-0.93	0.20	0.91	0.04	-0.97	0.22
	**ANX6**	**1.45**	0.04	**5.36**	0.22	**1.44**	0.05	**5.24**	0.24
	**ANX7**	0.78	0.03	***-3.61***	0.28	0.74	0.03	***-3.48***	0.26
									
**Underfit**	**>1.30**	1.00	**>2**	1.00	**>1.30**	1.00	**>2**	1.00	
**Overfit**	**<0.70**	0	**<-2**	2.00	**<0.70**	0	**<-2**	1.00	

		**Infit MNSQ**	**SE**	**Infit t**	**SE**	**Outfit MNSQ**	**SE**	**Outfit t**	**SE**

	**DEP1**	0.96	0.04	-0.74	0.20	0.88	0.04	-1.51	0.19
	**DEP2**	0.97	0.04	-0.03	0.25	0.98	0.08	0.17	0.23
	**DEP3**	0.86	0.04	-1.76	0.28	0.85	0.06	-0.93	0.29
	**DEP4**	1.17	0.03	**2.54**	0.21	1.11	0.03	1.71	0.19
	**DEP5**	1.12	0.06	1.26	0.27	1.20	0.11	1.06	0.21
	**DEP6**	0.73	0.03	***-3.82***	0.20	***0.64***	0.03	***-3.20***	0.19
	**DEP7**	1.17	0.06	1.41	0.25	**1.68**	0.19	**2.74**	0.19
									
**Underfit**	**>1.30**	0	**>2**	1.00	**>1.30**	1.00	**>2**	1.00	
**Overfit**	**<0.70**	0	**<-2**	1.00	**<0.70**	1.00	**<-2**	1.00	

**Table 3:** Fit statistics for PHQ-9 items (collapsed across sample size) for the Rating Scale Model

		**Infit MNSQ**	**SE**	**Infit t**	**SE**	**Outfit MNSQ**	**SE**	**Outfit t**	**SE**
	**PHQ1**	1.01	0.01	0.33	0.09	1.02	0.02	0.30	0.13
	**PHQ2**	0.72	0.01	***-4.04***	0.08	0.71	0.01	***-3.36***	0.07
	**PHQ3**	1.14	0.01	**2.39**	0.09	1.15	0.02	**2.25**	0.11
	**PHQ4**	0.87	0.01	***-2.09***	0.09	0.93	0.01	-0.82	0.09
	**PHQ5**	**1.37**	0.01	**4.19**	0.08	1.28	0.02	**2.46**	0.08
	**PHQ6**	1.10	0.01	1.27	0.08	0.95	0.02	-0.42	0.07
	**PHQ7**	1.04	0.01	0.60	0.08	0.86	0.01	-1.16	0.06
	**PHQ8**	1.25	0.02	**2.46**	0.08	0.97	0.02	-0.08	0.07
	**PHQ9**	1.17	0.03	1.48	0.10	0.84	0.05	-0.87	0.08
									
**Underfit**	**>1.30**	1.00	**>2**	3.00	**>1.30**	0	**>2**	2.00	
**Overfit**	**<0.70**	0	**<-2**	2.00	**<0.70**	0	**<-2**	1.00	

**Table 4:** Fit statistics for PHQ-9 items (collapsed across sample size) for the Partial Credit Model

	**Infit MNSQ**	**SE**	**Infit t**	**SE**	**Outfit MNSQ**	**SE**	**Outfit t**	**SE**
**PHQ1**	0.98	0.01	-0.02	0.09	0.96	0.02	-0.27	0.11
**PHQ2**	0.79	0.01	***-2.99***	0.07	0.74	0.01	***-3.19***	0.07
**PHQ3**	1.17	0.01	**2.95**	0.08	1.17	0.02	**2.55**	0.10
**PHQ4**	1.01	0.01	0.05	0.08	0.98	0.01	-0.25	0.08
**PHQ5**	1.22	0.01	**2.47**	0.08	**1.30**	0.03	1.93	0.09
**PHQ6**	0.95	0.01	-0.36	0.07	0.97	0.03	-0.26	0.08
**PHQ7**	0.91	0.01	-1.00	0.07	0.86	0.02	-1.04	0.07
**PHQ8**	1.05	0.02	0.34	0.08	0.95	0.03	-0.08	0.08
**PHQ9**	0.92	0.02	-0.22	0.06	1.00	0.10	-0.41	0.10
								
**>1.30**	0	**>2**	2.00	**>1.30**	0	**>2**	1.00	
**<0.70**	0	**<-2**	1.00	**<0.70**	0	**<-2**	1.00	

Tables 1 and 2 demonstrate that for both HADS subscales there was a broad agreement between the infit and outfit statistics. In other words, the numbers of items identified as misfitting were relatively consistent for the infit and outfit versions of the same type of statistic irrespective of the Rasch model applied.

However for the PHQ-9 (Tables 3 and 4) consistently more items were identified as misfitting by t-statistics (infit/outfit t-statistic) than by the equivalent mean square statistics. In terms of underfit to the model the Type I error rate for the t-statistics was at least double that of the corresponding mean square, e.g. for the Rating Scale Model, the total number of items exceeding the thresholds for infit t-statistic was 3, whereas for the infit mean square 1, and for the Partial Credit Model infit t-statistic it was 2, and the infit mean square 0. A similar pattern of results was also found for those items overfitting the models.

Finally, it can also be seen that the standard errors were uniformly smaller for the mean square statistics than those for the t-statistics, indicating greater levels of stability in the parameter estimates. This was particularly noticeable for the HADS, but also applied to some extent to the PHQ-9.

### 2. Fit Statistics – Sample Size

The relationship between sample size and fit statistics is shown in Tables 5, 6, 7, 8. This analysis has been broken down into overfitting (MNSQ < 0.7/t < -2) and underfitting/misfitting items (MNSQ > 1.3/t > 2).

**Table 5:** HADS – Rating Scale Model Error rates by sample size (collapsing across items)

**HADS A**	**Infit t**	**Infit t**	**Infit MNSQ**	**Infit MNSQ**	**Infit MNSQ**	**Outfit t**	**Outfit t**	**Outfit MNSQ**	**Outfit MNSQ**	**Outfit MNSQ**
	**<-2**	**>2**	**<0.7**	**>1.2**	**>1.3**	**<-2**	**>2**	**<0.7**	**>1.2**	**>1.3**

**25**	0	0	1	1	1	0	0	1	1	1
**50**	0	1	0	1	1	0	1	0	1	1
**100**	0	1	0	1	1	0	1	0	1	1
**200**	0	1	0	1	1	0	1	0	1	1
**400**	1	1	0	1	1	1	1	0	1	1
**800**	1	1	0	1	1	1	1	0	1	1
**1600**	5	1	0	1	1	4	1	0	1	1
**3200**	5	1	0	1	1	4	1	0	1	1

**HADS D**	**Infit t**	**Infit t**	**Infit MNSQ**	**Infit MNSQ**	**Infit MNSQ**	**Outfit t**	**Outfit t**	**Outfit MNSQ**	**Outfit MNSQ**	**Outfit MNSQ**

	**<-2**	**>2**	**<0.7**	**>1.2**	**>1.3**	**<-2**	**>2**	**<0.7**	**>1.2**	**>1.3**

**25**	0	0	0	2	2	0	0	1	2	1
**50**	0	0	0	2	2	0	0	1	1	1
**100**	0	0	0	2	2	1	0	1	2	2
**200**	0	2	0	2	2	1	0	0	2	0
**400**	1	2	0	2	1	1	1	0	1	1
**800**	1	2	0	2	2	2	1	1	1	1
**1600**	2	3	0	2	2	3	3	0	1	1
**3200**	2	3	0	2	2	3	3	0	1	1

The number of items exceeding the fit criteria are shown in each column

**Table 6:** PHQ9 – Rating Scale Model Error rates by sample size (collapsing across items)

	**Infit t**	**Infit t**	**Infit MNSQ**	**Infit MNSQ**	**Infit MNSQ**	**Outfit t**	**Outfit t**	**Outfit MNSQ**	**Outfit MNSQ**	**Outfit MNSQ**
**Sample**	**< -2**	**> 2**	**< 0.7**	**> 1.2**	**> 1.3**	**< -2**	**> 2**	**< 0.7**	**> 1.2**	**> 1.3**

**25**	0	0	0	0	2	0	0	0	0	2
**50**	0	0	0	1	0	0	0	0	0	1
**100**	0	1	1	0	1	0	0	1	0	1
**200**	1	1	0	0	2	1	0	0	0	0
**400**	1	3	0	0	1	1	1	0	0	0
**800**	2	3	1	0	1	1	2	2	0	0
**1600**	2	5	0	0	1	2	2	0	0	0
**3200**	2	5	0	0	1	4	2	0	0	0

**Table 7:** HADS – Partial Credit Scale Model Error rates by sample size (collapsing across items)

**HADS A**	**Infit t**	**Infit t**	**Infit MNSQ**	**Infit MNSQ**	**Infit MNSQ**	**Outfit t**	**Outfit t**	**Outfit MNSQ**	**Outfit MNSQ**	**Outfit MNSQ**
	**<-2**	**>2**	**<0.7**	**>1.2**	**>1.3**	**<-2**	**>2**	**<0.7**	**>1.2**	**>1.3**

**25**	0	0	0	1	1	0	0	1	1	1
**50**	0	1	0	1	1	0	0	0	1	1
**100**	0	1	0	1	1	0	1	0	1	1
**200**	0	1	0	1	1	0	1	0	1	1
**400**	2	1	0	1	1	2	1	0	1	1
**800**	3	1	0	1	1	3	1	0	1	1
**1600**	3	2	0	1	1	3	2	0	1	1
**3200**	4	2	0	1	1	4	2	0	1	1

**HADS D**	**Infit t**	**Infit t**	**Infit MNSQ**	**Infit MNSQ**	**Infit MNSQ**	**Outfit t**	**Outfit t**	**Outfit MNSQ**	**Outfit MNSQ**	**Outfit MNSQ**

	**<-2**	**>2**	**<0.7**	**>1.2**	**>1.3**	**>2**	**<-2**	**<0.7**	**>1.2**	**>1.3**

**25**	0	0	1	1	0	0	0	1	2	2
**50**	0	0	0	2	0	0	0	1	1	1
**100**	1	0	1	1	0	0	0	1	2	2
**200**	1	0	0	0	0	0	1	1	1	1
**400**	1	1	0	0	0	1	1	1	1	1
**800**	1	1	0	0	0	2	1	1	1	1
**1600**	2	3	0	0	0	2	2	1	2	1
**3200**	3	3	0	0	0	3	3	1	2	1

**Table 8:** PHQ9 – Partial Credit Scale Model Error rates by sample size (collapsing across items)

	**Infit t**	**Infit t**	**Infit MNSQ**	**Infit MNSQ**	**Infit MNSQ**	**Outfit t**	**Outfit t**	**Outfit MNSQ**	**Outfit MNSQ**	**Outfit MNSQ**
**Sample**	**< -2**	**> 2**	**< 0.7**	**> 1.2**	**> 1.3**	**< -2**	**> 2**	**< 0.7**	**> 1.2**	**> 1.3**

**25**	0	0	0	2	0	0	0	0	2	2
**50**	0	0	0	1	0	0	0	0	1	1
**100**	0	0	0	2	1	0	0	0	2	2
**200**	0	0	0	1	0	0	0	0	0	0
**400**	1	2	0	2	0	1	1	0	2	0
**800**	1	2	0	2	0	1	2	0	2	0
**1600**	1	2	0	1	0	2	2	0	2	0
**3200**	2	2	0	1	0	2	2	0	2	0

#### Overfitting items

It can be seen that for both the infit and outfit mean squares few items were identified with mean square fit statistics < 0.7 for the HADS subscales (Tables 5 and 7) and the PHQ-9 (Tables 6 and 8). In contrast to this, the corresponding t-statistics (< -2) demonstrated that as sample size increased, the number of items identified as misfitting rapidly increased. For instance, for the Rating Scale Model, the infit mean squares for HADS-D (Table 5) consistently failed to identify a single instance of an item misfitting as sample size increased, whereas the corresponding t-statistic identified no misfit between sample sizes 25 and 200. Furthermore, there was only 1 instance of misfit at sample sizes of 400 and 800, and 2 instances at sample sizes of 1600 and beyond. This pattern was particularly evident for the HADS-A Partial Credit Model (Table 7). A similar pattern was also observed for the PHQ-9 (Table 8).

#### Underfitting items

There was a clear link observed between sample size and fit statistic when comparing infit and outfit mean squares above 1.2 with t-statistics > +2. Once again, t-statistics increased in proportion to sample size, whereas the mean square equivalents remained approximately invariant to sample size changes (compare for instance the infit statistics on the Rating Scale for HADS-D in Table 5, as well as for the PHQ-9 in Table 6). Additionally, more instances of misfit were identified, in general, when a mean square of >1.2 was used compared with 1.3, although this was not always consistently the case.

### 3. Fit Statistics, Sample size and individual items

#### Items not demonstrating misfit

In terms of agreement between the four statistics for individual items not exhibiting misfit it can be seen from Table 1 that, for instance for HADS-A, the infit and outfit means squares agreed with their equivalent t-statistic on 5 items for the Rating Scale Model; similarly there was agreement between the fit statistics for 4 items from the HADS-D. Slightly less consistency was observed for both subscales on the Partial Credit Model (Table 2) and for both models using the PHQ-9, although again there was agreement for the majority of items (Tables 3 and 4).

**Figure 1:**
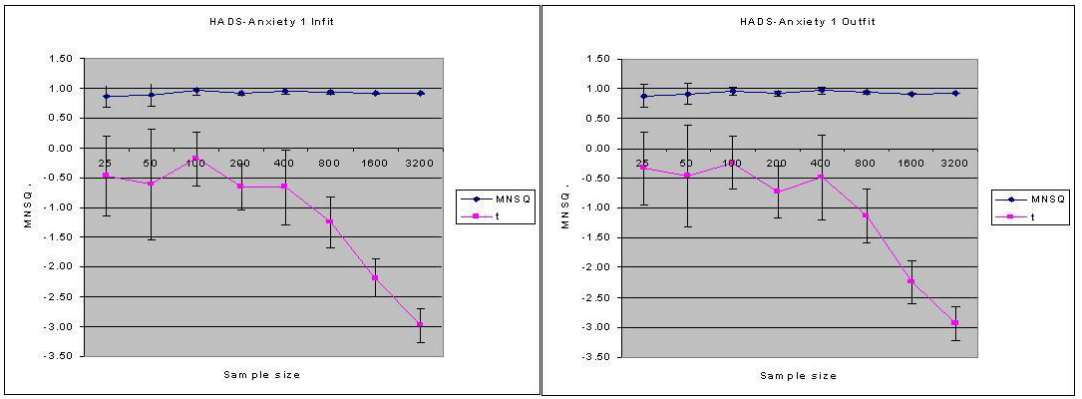
Infit and Outfit statistics by sample size for HADS-Anxiety 1.

An example of an item (HADS-A1) which had demonstrated fit across all four statistics is shown in Figure 1. Although Table 1 demonstrated fit for the t-statistics it can be seen that whereas the item demonstrated consistent (infit and outfit) mean square statistics (approx. 0.92) across sample size, the infit and outfit t-statistics became increasingly more negative as sample size increased (> 200), resulting in the t-statistics highlighting significant overfit for this item at sample sizes greater than 1600.

#### Overfitting items

**Figure 2:**
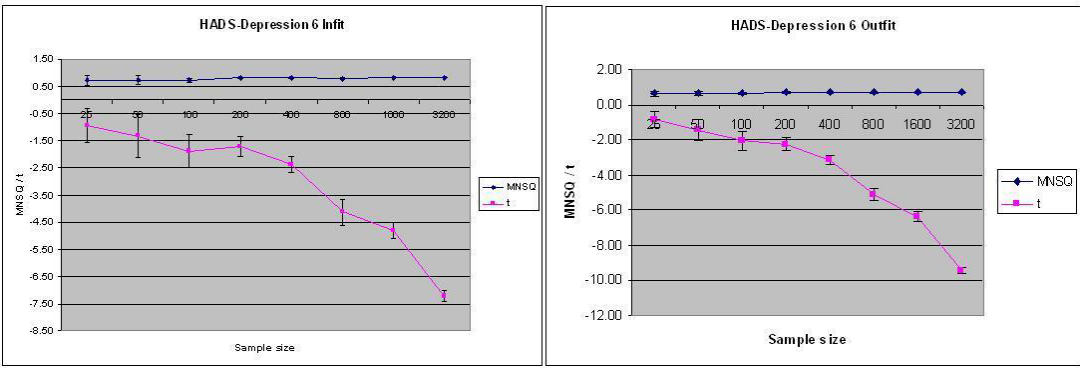
Infit and Outfit statistics by sample size for HADS-Depression 6.

For the Rating Scale Model one HADS-A item (7) and one HADS-D item (6), as well as two PHQ-9 items (2 and 4) were identified as overfitting by the t-statistics but not by the mean squares. The Partial Credit Model demonstrated overfit for HADS-D6, as well as HADS-A3, and PHQ2. Figure 2 demonstrates once again that whereas the mean squares remained consistent across sample size, the t-statistics became increasingly more negative (sample size > 200).

#### Underfitting items

**Figure 3:**
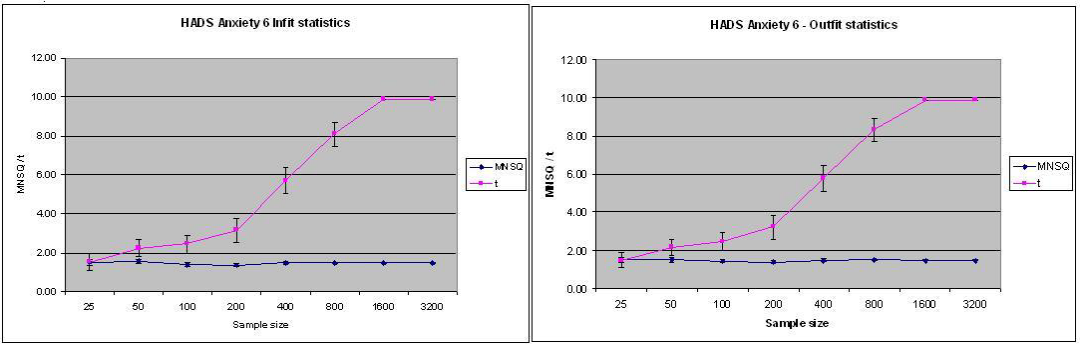
Infit and Outfit statistics by sample size for HADS-Anxiety 6.

HADS-D5 and HADS-D7 were identified as underfitting on the Rating Scale Model (RSM) for both the in- and outfit t-statistics, but not the mean squares, although neither was identified as misfitting on the Partial Credit Model (PCM); HADS-D4 was identified as misfitting (i.e. underfitting) on the Partial Credit Model by the infit t alone. PHQ3 and 5 were identified as misfitting by both infit and outfit t-statistics for the RSM and PCM, but not by the mean squares. Finally, HADS-A item 6 was consistently identified as misfitting (underfitting) by all four statistics, yet when the four statistics are plotted against sample size (Figure 3) it is apparent that this item was only identified by the t-statistics as under/misfitting once the sample size exceeded 200.

In summary, two instances of misfit for the t-statistics could be discerned from the data: 1). instances where mean square statistics fell within the critical (0.7 – 1.3) range (i.e. "fit"), and 2). instances where mean square statistics fell outside this range, in particular exceeding 1.3 (misfit).

Items identified as falling within range (0.7 – 1.3) showed consistent mean squares (infit/outfit) as sample size increased; on the other hand, the corresponding t-statistics increased with sample size (i.e. identified misfit where none was identified as such by the corresponding mean square). Items identified as falling outside the critical range (0.7 – 1.3) were consistently identified as misfitting by mean squares, but only identified as such by the corresponding t-statistics when sample size exceeded 200. Beyond these limits t-statistics increased in proportion with sample size. In other words, the t-statistic only identified items as misfitting for larger sample sizes.

## Discussion

The aim of this study was to explore the relationship between sample size and four commonly used fit statistics for two polytomous Rasch Models. The results of this study demonstrated that Type I error rates – defined strictly in this study as falsely rejecting an item as not fitting the Rasch model – for the t-statistic were at least twice those of the corresponding fit statistic for both infit and outfit for both Rasch Models. In addition, the results of the analysis of sample size and fit statistic suggested that whereas the mean square fit statistics broadly remained constant in the number of items whether identified as fitting or misfitting (under and over), the instances of misfit identified by the t-statistics increased proportionally with sample size. Further analysis of the individual item fit and sample size suggested that although in the majority of cases there was agreement between mean square and t-statistics in terms of identifying fit and misfit (>50% for both models and instruments), there were discrepancies in Type I error rate as defined in this study and a lack of sample size invariance for the t-statistics.

The results of the study suggest that t-statistics are highly dependent on sample size which has the effect of inflating putative Type I error rates. Specifically, for cases where mean square statistics fell within the range 0.7 – 1.3, the t-statistics increased in magnitude as sample size increased, therefore for the t-statistic the Type I error rate was inflated and the probability of identifying misfit where none was identified by the mean square statistics increased with sample size. Similarly, where mean square statistics identified misfit outside the 0.7 – 1.3 range, t-statistics only identified misfit as the sample size increased to beyond 200.

In terms of Type I error rates, for Rating Scale Model the outfit mean square statistics provided the most stable rates, whereas the infit mean squares were more stable for the Partial Credit Model, although there was little difference in identifying misfit between the 1.2 and 1.3 criteria for mean squares.

Taken together these results suggest that both infit and outfit mean square statistics are relatively insensitive to sample size variation for polytomous data, and that t-statistics may vary considerably with sample size. The latter has confirmed previously reported findings using simulated data sets [15].

The potential cause for this sample size dependence for the t-statistics may lie with the standard deviations. The results of previous research have demonstrated that the variability of the mean squares decreases significantly [19] by sample size. As the t-statistics are derived from the mean squares and their standard deviations it appears that t-statistics are disproportionately affected by decreases in variability. The fact that t-statistics are highly dependent on the variance and thereby sample size has also been demonstrated in previous studies with the dichotomous model [15].

Although the results for the t-statistics confirm results from previous studies (e.g. "Knox Cube Test") [15] they differ markedly from existing literature on simulated data using the dichotomous model [15,16] which, in addition, has also suggested a significant sample size dependence for mean square statistics. For instance, Karabatsos [15] generated data sets with sample sizes of 150, 500 and 1000 and test sizes of 20 and 50 items. Ability, θ, was distributed as N(0, 1) and item difficulty, δ, as U(-2 to +2). Type I error rates were evaluated for both infit and outfit at critical values: 1.1, 1.2 and 1.3. The results indicated both fit statistics were clearly a function of sample size, and test length to a lesser extent.

This gives rise to a potential discrepancy between the dichotomous and polytomous Rasch Models and Type I error rates, suggesting a dependence between sample size and fit for the dichotomous model for both mean square and t-statistics, in contrast to sample size independence for mean square fit statistics for the polytomous model as demonstrated in this study, and further research will be required to elucidate this issue.

There are a number of limitations to this study: 1). The primary limitation is that "real" data directly derived from patients were used rather than simulated data. Previous work on the HADS in particular had demonstrated the presence of misfitting items in the scale [6]. The aim was to observe how effectively the four fit statistics identified misfit and whether and to what extent this was affected by sample size. However, we acknowledge that estimates of true Type I error rates are more optimally derived from simulated data where fit and misfit may be artificially manipulated. Further limitations reflect the fact that the data were restricted to cancer patients only, and only included mental health questionnaires. Additionally, the relationship between sample size and instrument length was not explored, although there were modest differences in test length between the HADS and PHQ-9. Finally any potential interactions with dimensionality and item difficulty [15] were also not explored.

The presence of underfitting items in instruments may have a potentially significant impact by severely degrading the measures, whereas overfitting items will tend to overestimate differences in raw scores [11]. The former may lead to an under-detection of health problems (e.g. low levels of screening efficacy), the latter may interfere in comparisons within and between individuals. Clearly the need to accurately identify misfitting, particularly underfitting items is paramount. This study demonstrated that low Type I error rates were evidenced by mean square fit statistics, which appeared independent of sample size. The clinical impact of erroneously removing misfitting items has not been directly investigated, however research suggests that the converse problem of retaining misfitting items (Type II errors) has little or no impact on the efficacy of, for instance, instruments used to screen for psychological distress [6]. Research on both the HADS [6] and the Geriatric Depression Scale [28] suggests that misfitting items may be removed from the instruments whilst maintaining, if not improving screening efficacy (in terms of diagnosing cases of anxiety or depression) when compared with a gold standard psychiatric interview. Although the clinical implications of Type I and II errors needs to be explored further, the results suggest that correctly identifying misfit has a direct benefit to patients by reducing the burden of the number of questions needing to be answered (whilst maintaining efficacy of the instrument).

## Conclusion

In summary, the study suggests that for polytomous Rasch Models when evaluating against accepted threshold criteria the t-statistics are sample size dependent. In contrast to this sample size invariance appears to exist for the mean square fit statistics. It may therefore be recommended that t-statistics should be adjusted or interpreted with caution when judging item fit or attempting to identify misfit in data, particularly for large samples and polytomous data.

## Competing interests

The authors declare that they have no competing interests.

## Authors' contributions

LJF, GV and MS contributed the data for this study. The data analysis was performed by ABS and RR. The manuscript was drafted by ABS with critical comments provided by RR, LJF, GV and MS. All authors read and approved the final manuscript.

## Pre-publication history

The pre-publication history for this paper can be accessed here:



## Supplementary Material

Additional file 1 The file format is in MS Word, and is entitled "Appendix 1". It contains five formulae with descriptions. Click here for file
