# Supplementary material for: Rasch fit statistics and sample size considerations for polytomous data
Source: BMC Med Res Methodol. 2008 May 29;8:33. doi: 10.1186/1471-2288-8-33 (PMC2440760; doi:10.1186/1471-2288-8-33)
Supplement: Additional file 1 — The file format is in MS Word, and is entitled "Appendix 1". It contains five formulae with descriptions. [file 1471-2288-8-33-S1.doc]

Appendix 1

The equations (derived from Bond & Fox, 2001) for the mean square and t fit statistics are presented below.

For the mean square statistics *Z_ni_* is the standardised residual variance for each item/person interaction; W_ni_ is the individual residual variance.

1. Unweighted (Outfit) Mean Square:

2. Weighted (Infit) Mean Square:

3. Infit / Outfit t (ZSTD):

The infit / outfit mean squares can be converted to t statistics using the Wilson-Hilferty* formula, where MS refers to the mean square statistic, and S is the variance.

$$t=\left[\left(MS^{1/3}-1\right)\left(3/S)+\left(S/3\right)\right)\right]$$

*Wilson, E. B., & Hilferty, M. M. The distribution of chi-square. *Proc Natl Acad Sci USA* 1931; **17**: 684-688.

4. Rating Scale Model (Andrich, 1978)

or expressed as log-odds (logits),

5. Partial Credit Model (Masters, 1982)

, expressed as log-odds,
